# Gender Differences in the Symptoms, Signs, Disease History, Lesion Position and Pathophysiology in Patients with Pulmonary Embolism

**DOI:** 10.1371/journal.pone.0133993

**Published:** 2015-07-24

**Authors:** Xingqi Deng, Yanyan Li, Ling Zhou, Chunyan Liu, Mei Liu, Nianchang Ding, Jinyan Shao

**Affiliations:** Department of Emergency Medicine, the Center Hospital of Minhang District, Fudan University, Shanghai, China; National Institute for Viral Disease Control and Prevention, CDC, CHINA

## Abstract

Advances in research relating to pulmonary embolisms (PE) can assist physicians in selecting the best management strategies for PE patients. However, the symptoms, signs, disease history, lesion position and pathophysiology linked to different genders in patients with PE have rarely been evaluated. One hundred and forty-nine PE patients (73 males and 76 females) were sequentially recruited to this study over the last five years whilst attending our Emergency Department. Data relating to the symptoms, signs, disease history, biochemical testing, cardiac electrophysiology, imaging detection, treatment and outcome were collected and the gender differences were analyzed. We found that embolisms occurred significantly more frequently in the right lung (89.7%) than in the left lung (42.6%). The presence of dyspnea, the number of patients presenting with tumors, the number of patients with chronic pulmonary disease, those with emboli in the right pulmonary artery and emboli in the right lung, as well as the average systolic and diastolic blood pressure were: 78.1%, 15.1%, 31.5%, 32.9%, 94.5%, 129.9+20.0 and 75.0+11.2 in the male patients and 59.2%, 1.3%, 14.5%, 17.1%, 69.7%, 125.1+14.6 and 69.3+11.0 in the female patients. These indicators were found to be significantly higher in male patients. In contrast, the rate of V1-V4 T-wave inversion and level of D-dimer in the blood were significantly lower in males than in females. No significant difference was observed in the remaining observational indicators. Gender differences regarding the symptoms, signs, disease history, lesion position and pathophysiology exist in patients with PE and should be considered in clinical practice.

## Introduction

Pulmonary embolism (PE) is a serious multiple-organ-involved disease commonly originating from a deep venous thrombosis (DVT), with a morbidity of approximately 69 per 100,000 people [1]. Patients who have been treated for PE have an 8% mortality rate, whereas untreated PE patients have a mortality rate as high as 30%, according to a previous study performed around 20 years ago [2]. PE can be broadly classified as either massive or submassive [3]. Patients with submassive PE can be treated with anticoagulation medication alone as they are generally hemodynamically stable, whilst patients with massive PE usually present with hemodynamic instability and are treated with either pulmonary embolectomy or thrombolytic therapy [4].

The major risk factors for the development of PE include intrinsic factors such as previous venous thromboembolism and age >70 years, and acquired factors such as malignancy, cancer chemotherapy, paralysis, major or lower limb trauma, lower limb orthopedic surgery, general anesthesia for >30 minutes, heparin-induced thrombocytopenia and antiphospholipid antibodies [5]. Other minor risk factors for PE are an inherited hypercoagulable state, obesity, pregnancy or puerperium, estrogen therapy, prolonged immobility, nephrotic syndrome, etc. [6]. Although these risk factors are important for PE control and prevention in clinical practice, about a quarter of patients with PE have no apparent provoking risk factor, half have a temporary provoking risk factor such as a history of PE and recent surgery, and a quarter have complications from various cancers [5, 6].

The diagnosis of PE is a relatively complex and rigorous process because ‘confirmed PE’ indicates the need for PE-specific treatment and ‘excluded PE’ justifies the validity of withholding such treatment [7]. To establish a PE diagnosis, the following symptoms, signs, history and medical examinations must be considered: clinical presentation, such as dyspnea, chest pain, cough, hemoptysis, syncope, tachypnea, tachycardia, fever, etc. [7]; assessment of clinical probability using prediction rules such as the Wells score and the revised Geneva score [8]; elevated D-dimer (a degradation product of cross-linked fibrin) level in plasma [9]; evidence from compression ultrasonography and computed tomographic venography; evidence from ventilation–perfusion scintigraphy; evidence from computed tomography; pulmonary angiography [7–10], etc.

Recently, research in the epidemiology, predisposing factors, natural history and pathophysiology of PE have been advancing greatly [4, 7]. These studies have helped to improve the diagnosis, treatment, and prognosis significantly [4, 7]. The acute case fatality rate for PE now ranges from 7 to 11% according to a prospective cohort of studies and is continuing to decrease [11]. Nevertheless, many issues regarding the natural history and pathophysiology of PE require further study. Any advances made regarding the above issues will facilitate selection of the best management strategies for a typical patient suffering from a given condition, taking into account the impact on outcome, as well as the risk/benefit ratio of particular diagnostic or therapeutic means [12]. PE can be difficult to diagnose as the clinical signs and symptoms are non-specific. Thus, further research on the relative symptoms, signs, disease history and pathophysiological characteristics of PE is still important. Questions that still need addressing include: Do any differences exist in the distribution of lesions within the lungs of PE patients? How do the demographic characteristics, symptoms, signs, disease history and phenotypes of pathophysiology differ between male and female patients? In this report, 149 PE patients were recruited at our Emergency Department from January 2010 to December 2014 to evaluate the above questions.

## Materials and Methods

### Patients

This study was conducted in accordance with the World Medical Association Declaration of Helsinki. The Review Board of the Ethics Committee of Medical Research at the Center Hospital of Minhang District (170 Xinsong Road, Minhang District, Shanghai 201199, China) approved the study protocols (reference number: SHMHCH 2010–002). Written informed consent was obtained from all patients according to the guidelines of the Chinese National Ethics Regulation Committee; the procedure was explained to all patients and we emphasized that their data would be used in this study. All patients were informed of their rights to withdraw consent personally or via kin, caretakers, or guardians.

We sequentially recruited 149 PE patients treated in our hospital Emergency Department from January 2010 to December 2014. The diagnoses complied with the “2014 ESC Guidelines on the diagnosis and management of acute pulmonary embolism” [12]. Patients presenting with PE symptoms and signs were screened by clinical examination and assessment of clinical probability, in combination with other tests at the emergency hospital phase. For suspected PE with shock or hypotension, computed tomographic pulmonary angiography (CTPA) was first adopted to confirm PE. For CTPA-negative patients, echocardiography was then performed to provide evidence of any acute pulmonary hypertension or right ventricle dysfunction.

For those suspected PE patients presenting without shock or hypotension, the clinical probability of PE was assessed using clinical judgment or a prediction rule [12]. Patients with high clinical probability of PE had their diagnosis confirmed by CTPA. Plasma D-dimer measurements were performed on patients with a low or intermediate clinical probability of PE. CTPA was used to confirm the diagnosis of PE in patients with elevated D-dimer levels (>250 ng/mL D-Dimer Units).

For patients in whom both CTPA and echocardiography were negative, the differential diagnosis would then include consideration of acute valvular dysfunction, tamponade, acute coronary syndrome (ACS) and aortic dissection. Ancillary bedside imaging tests including transesophageal echocardiography and bedside compression venous ultrasonography as well as catheterization would be performed.

To survey the mortality of the PE patients treated in our hospital, the death cases during the three-month follow-up period were recorded and the death rate was calculated.

### Data collection

The questionnaire explored demographics (age and gender); symptoms (dyspnea, cough, palpitation, chest pain, fever, syncope and hemoptysis); and personal history (PE, tumor, heart disease, chronic pulmonary disease, alcohol consumption, cigarette smoking, obesity, hypertension, diabetes and cerebrovascular cardiovascular disease). Any data collected via the questionnaire was confirmed by in-hospital measurement when the relevant assessment method became available.

Physical signs in each patient, including engorgement of the neck veins, edema of the lower extremities, respiratory rate, systolic pressure, diastolic pressure, heart rate and cardiac sounds, were examined and collated at the Emergency Department registry.

To identify any complications in the cardiovascular system, a 12-lead surface electrocardiogram was performed in the Emergency Department. As well as the structure and dysfunction of the heart, the following were recorded: P-pulmonale, right bundle branch block (RBBB), V1–V4 T-wave inversions, a large S wave in lead I, a large Q wave in lead III, an inverted T-wave in lead III (S1Q3T3), atrial fibrillation, pulmonary arterial hypertension, tricuspid insufficiency, and left ventricular ejection fraction (LVEF). The chief technician reviewed the final electrocardiographic and echocardiographic results for each patient. Any uncertainties regarding the electrocardiographic and echocardiographic results were resolved by discussion between the technicians. Both technicians who reviewed these data were blinded to the overall clinical data and group division.

In order to screen for pneumonia, pleural effusion, and heart shadow changes, an X-ray examination was performed immediately after the patient attended the Emergency Department.

For all suspected PE patients, CTPA was performed to reveal the position and extent of any lung injury caused by the embolism. The position of the embolism was then categorized as follows: right pulmonary artery; left pulmonary artery; upper lobe, middle lobe and lower lobe of the right lung; and upper lobe and lower lobe of the left lung.

Blood gas analysis revealed the following biochemical results: D-dimer, creatinine, lactate dehydrogenase, creatine kinase, brain natriuretic peptide, etc. These were closely monitored during and after the emergency rescue process.

### Statistical analysis

Continuous variables were presented as means ± standard deviation (SD) and categorical data were presented as numbers (percentage). Differences between female and male groups were examined by using t-tests or χ^2^ tests according to the characteristics of the data distribution. The significance level (α) was set at 0.05. All statistical analyses were performed using Stata/SE 12.0 for Windows (StataCorp LP).

## Results

### Clinical characteristics of PE patients

In total 149 patients with PE were recruited for this report, of whom, 73 were males and 76 were females. The male: female constituent ratio was close to 1.0 and is coincident with other reports (Table 1) [13]. The average age of the 149 PE patients was 73.5±13.4 years, with most patients tending to be older. The overall fatality rate was 7.4% (Table 1). Severe pneumonia, cardiogenic shock, hemodynamic compromise and/or respiratory failure were found to be the main causes of death.

**Table 1:** Comparison between male and female patients.

	Male (n = 73)	Female (n = 76)	Total (n = 149)	*P* value
*Demography*				
Age	72.6±14.7	75.0±15.1	73.5±13.4	*0*.*086*
*Symptom*				
Dyspnea	57 (78.1)	45 (59.2)	102 (68.5)	***0*.*021***
Cough	51 (69.9)	41 (53.9)	92 (61.7)	*0*.*067*
Palpitation	25 (34.2)	28 (36.8)	53 (35.6)	*0*.*862*
Chest pain	16 (21.9)	7 (9.2)	23 (15.4)	*0*.*055*
Fever	19 (26.0)	15 (19.7)	34 (22.8)	*0*.*471*
*Sign*				
Syncope	6 (8.2)	12 (15.8)	18 (12.1)	*0*.*244*
Hemoptysis	5 (6.8)	2 (2.6)	7 (4.7)	*0*.*269*
Respiratory rate	20.0±1.9	20.0±2.7	20.0±2.1	*0*.*417*
Systolic pressure	129.9±20.0	125.1±14.6	126.2±15.6	***0*.*048***
Diastolic pressure	75.0±11.2	69.3±11.0	72.3±14.1	***0*.*001***
Engorgement of the neck veins	23 (31.5)	20 (26.3)	43 (28.9)	*0*.*603*
Heart rate	91.9±11.3	91.3±17.7	91.5±12.4	*0*.*416*
Edema of lower extremity	21 (28.8)	15 (19.7)	36 (24.2)	*0*.*273*
*Disease history*				
Tumor	11 (15.1)	1 (1.3)	12 (8.1)	***0*.*005***
Heart diseases	25 (34.2)	27 (35.5)	52 (34.9)	*1*.*000*
Chronic pulmonary disease	23 (31.5)	11 (14.5)	34 (22.8)	***0*.*023***
PE or DVT history	5 (6.8)	8 (10.5)	13 (8.7)	*0*.*617*
Immobilization and/or surgery	24 (32.9)	38 (50.0)	62 (41.6)	*0*.*051*
*Blood gas analysis*				
PO2 (mmHg)	87.2±41.4	96.3±45.9	89.2±42.8	*0*.*122*
PaCO2 (mmHg)	39.9±11.1	37.6±10.1	38.4±9.8	*0*.*106*
pH	7.43±0.05	7.44±0.08	7.43±0.05	*0*.*109*
*Electrocardiogram*				
P-pulmonale	3 (4.1)	3 (3.9)	6 (4.0)	*1*.*000*
RBBB	8 (10.9)	8 (10.5)	16 (10.7)	*1*.*000*
V1–V4 T wave inversion	7 (9.6)	19 (25.0)	26 (17.4)	***0*.*024***
Atrial fibrillation	10 (13.7)	19 (25.0)	29 (19.5)	*0*.*124*
*Imaging tests*				
Pneumonia	39 (53.4)	34 (44.7)	73 (49.0)	*0*.*371*
Pleural effusion	28 (38.4)	29 (38.2)	57 (38.3)	*1*.*000*
Increased heart shadow	20 (27.4)	23 (30.3)	43 (28.9)	*0*.*841*
*Biochemical tests*				
D-dimmer (nmol/L)	7.9±10.3	13.6±16.6	11.8±9.7	***0*.*012***
Creatinine	77.6±100.8	60.7±41.9	69.9±89.4	*0*.*098*
Lactate dehydrogenase	458.0±787.4	368.4±309.4	436.0±387.0	*0*.*185*
Creatine kinase	192.2±250.1	190.3±260.0	191.5±244.1	*0*.*483*
Brain natriuretic peptide	2587.9±5362.9	4338.5±11922.9	3989.4±7974.2	*0*.*150*
*Outcome*				
Death	6 (8.2)	5 (6.6)	11 (7.4)	*1*.*000*

Continuous variables were presented as means ± standard deviation (SD) and categorical data were presented as the number (percentage). Differences between female and male groups were examined by using T test or χ^2^ tests according to the characteristics of data distribution. PE, pulmonary embolism; DVT, deep vein thrombosis; PO2, oxygen partial pressure; PaCO2, partial pressure of carbon dioxide; RBBB, right bundle branch block.

### Comparison between male and female PE patients

To identify differences between the male and female PE patients regarding age, symptoms, signs, disease history, pathophysiology and outcome, the patients were divided into male and female groups. As shown in Table 1, there was no significant difference between male and female PE patients in the following variables. Symptom: presence of cough, palpitation, chest pain, or fever; Sign: syncope, hemoptysis, engorgement of the neck veins and edema of lower extremities, respiratory rate, heart rate; History: history of heart disease, PE or DVT history, history of immobilization and/or surgery; blood gas analysis: PO2, PaCO2, pH; electrocardiogram: rates of P-pulmonale, RBBB, V1–V4 T-wave inversion and atrial fibrillation; imaging tests: presence of pneumonia, pleural effusion and increased heart shadow; biochemical tests: levels of blood creatinine, lactate dehydrogenase, creatine kinase and brain natriuretic peptide; outcome: rates of recovery and death.

It is noteworthy that many of these variables, such as the presence of cough, chest pain, and a history of immobilization and/or surgery, displayed different tendencies between male and female patients that were not considered to be significant because of the relatively small sample size (Table 1).

Interestingly, whilst the number of patients with dyspnea, tumor and chronic pulmonary disease; systolic and diastolic pressures, was significantly higher in males than in females, the number of patients with V1–V4 T-wave inversion and elevated blood D-dimer levels was significantly lower in males than in females (Table 1).

### PE lesions are more common in the right lung

**Fig 1:**
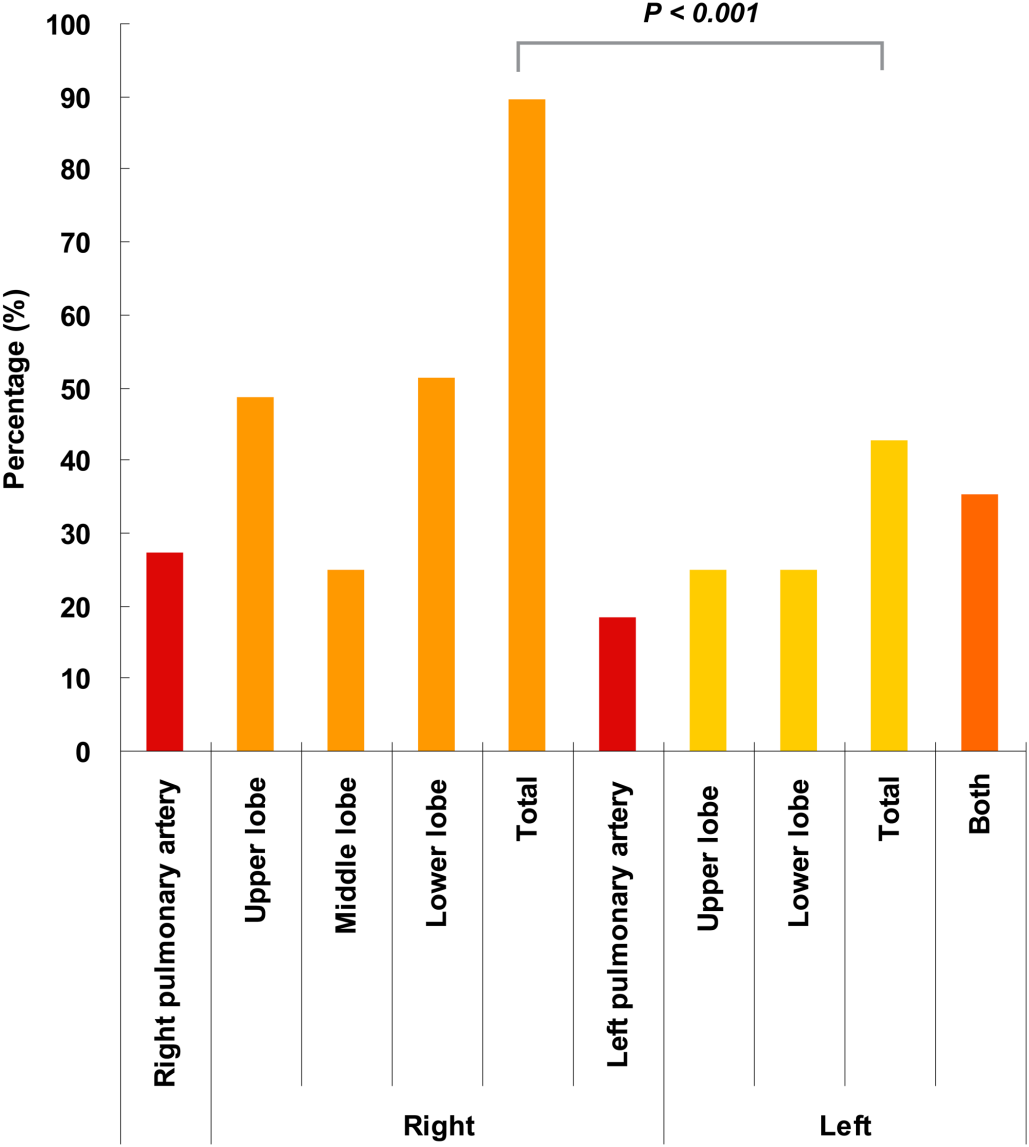
PE locations in the lung. The PE locations confirmed by computed tomographic pulmonary angiography (CTPA) were calculated. Lesions caused by PE were generalized as right pulmonary artery, right lung (upper, middle and lower lobes), left lung (upper and lower lobes) and left pulmonary artery. * Frequency of lesions located in the right lung is significantly higher than that in the left lung (P<0.001).

To evaluate differences in the locations of PE, the data from all patients with confirmed PE using CTPA were analyzed further. As shown in Fig 1, the numbers of PE located in the right pulmonary artery, right upper lobe, right middle lobe, right lower lobe, left pulmonary artery, left upper lobe and left lower lobe were 27.2%, 48.5%, 25.0%, 51.5%, 18.4%, 25.0% and 25.0%, respectively. The total proportion of right lung emboli (i.e., any confirmed embolism regardless of whether it was sited in a blood vessel or lung lobe) was 89.7%, which was significantly higher than that of the left lung (42.6%).

### Comparison of the PE location in males and females

The PE location in male patients with confirmed PE was compared with that of female patients. As shown in Table 2, the number of PEs located in the right pulmonary artery, left pulmonary artery, right lung, left lung, both lungs and right lung only was 32.9%, 21.9%, 94.5%, 38.4%, 34.2% and 60.3% in males, respectively; and 17.1%, 11.8%, 69.7%, 39.5%, 28.9% and 40.8%, respectively, in females. The numbers of PE lesions in the right pulmonary artery and right lung were significantly higher in male than in female patients (Table 2).

**Table 2:** Differences of the PE location between males and females.

PE location	Male (n = 73)	Female (n = 76)	*P* value
Right pulmonary artery	24 (32.9)	13 (17.1)	***0*.*0416***
Left pulmonary artery	16 (21.9)	9 (11.8)	*0*.*1542*
Right lung	69 (94.5)	53 (69.7)	***0*.*0002***
Left lung	28 (38.4)	30 (39.5)	*1*.*0000*
Both lungs	25 (34.2)	22 (28.9)	*0*.*6033*
Right lung only	44 (60.3)	31 (40.8)	***0*.*0269***

Categorical data were presented as the number (percentage). Differences between female and male groups were examined by using χ^2^ tests.

## Discussion

Differences relating to sex and gender are observed in the pathophysiological processes of many diseases [14–16]. In this study, we found differences according to sex in the symptoms, signs, disease history, hemodynamic heart consequences, biochemical indices, and the location of lung lesions in PE patients. Firstly, the frequency of right lung PE lesions was significantly higher than in the left lung generally. Secondly, our data suggested that the probability of PE lesions in the right pulmonary artery and/or right lung was significantly higher in male patients than in female patients. Thirdly, the occurrence of dyspnea, number of patients with tumor, number of patients with chronic pulmonary disease, and the average systolic and diastolic pressure were significantly higher in males than in females. Finally, in contrast with this, the rates of V1–V4 T-wave inversion and elevated D-dimer blood levels were significantly lower in males than in females.

Differences according to sex and gender in the pathophysiological procedure of PE have also been reported. Males were found to have lower survival rates than females [17]. Normotensive female PE patients were on average older and had a submassive PE stadium more frequently [18]. Changes in the right ventricle function over time during the course of a 3-month follow-up might differ between male and female patients with acute pulmonary thromboemboli, and the recovery process could be slower in females [19]. As far as we are aware, few previous reports have concerned the above four issues, and we have therefore addressed them in our study. Females hospitalized with PE were found to have significantly lower odds of 30-day mortality compared with males [20]. Of the PE patient population in our study, the death rate of male and female patients was 8.2% and 6.6% respectively; however, this difference was not significant.

The reason that PE lesions are more prevalent in the right lung is unknown. Anatomic characteristics and lung circulation might be fundamental factors for this phenomenon. Asthma, chronic obstructive pulmonary disease, chronic bronchitis, emphysema, pneumonia, lung cancer and acute respiratory distress syndrome are the most common diseases that target the lung, but it is difficult to find evidence that these diseases are also more prevalent in the right lung. Thus while our findings are useful in the management of PE, the importance of checking for this phenomenon in other lung diseases should also be emphasized.

Right ventricular dilatation and dysfunction are common complications of PE because of increased right ventricular after-load. These complications may lead to right ventricular failure and subsequently death [7, 12]. Echocardiograms are essential in ruling out complications such as these, and also intracardiac thrombi. In this study, although an echocardiogram was not performed on all patients at the Emergency Department, the electrocardiogram and imaging tests did show pathologic changes in the heart. These pathologic changes included increased heart shadow, P-pulmonale, RBBB, V1–V4 T-wave inversion and atrial fibrillation. As these tests are not specific to PE-related heart dysfunction, and we had no background information regarding the heart function of these patients, whether the above observations are specific consequences of PE is unknown. The reasons for patient death in this study were advanced age and missed hospital treatments.

PE is difficult to diagnose and may therefore be missed because of non-specific clinical presentation. However, early diagnosis is fundamental, since immediate treatment is highly effective. Depending on the clinical presentation, initial therapy is aimed primarily at either life-saving restoration of blood flow through occluded pulmonary arteries or the prevention of potentially fatal early recurrences. Thus, the results presented in this report will complement current strategies in PE control and treatment.

All patients in this report had CTPA data; embolism lesions were found in as many as 90% of PE patients who underwent CTPA at our hospital, which is considerably higher than noted in other reports [7, 12]. In this study, less than 10% of patients displayed shock or hypotension at the time of attending the hospital, which might be a result of local public health policies.

## Conclusions

Gender differences regarding the symptoms, signs, disease history, lesion position and pathophysiology exist in patients with PE and should be considered in clinical practice.
